# Mass photometry reveals SARS-CoV-2 spike stabilisation to impede ACE2 binding through altered conformational dynamics†

**DOI:** 10.1039/d2cc04711j

**Published:** 2022-10-28

**Authors:** Sean A. Burnap, Weston B. Struwe

**Affiliations:** Physical and Theoretical Chemistry Laboratory, Department of Chemistry, University of Oxford South Parks Road OX1 3TA UK weston.struwe@chem.ox.ac.uk; The Kavli Institute for Nanoscience Discovery, Dorothy Crowfoot Hodgkin Building South Parks Road OX1 3QU UK

## Abstract

Here we show using mass photometry how proline substitutions, commonly used for SARS-CoV-2 spike stabilisation in vaccine design, directly affects ACE2 receptor interactions *via* dynamics of open and closed states. Conformational changes and ACE2 binding were influenced by spike variant and temperature, but independent of site-specific *N*-glycosylation.

## Abstract 2

Here we show using mass photometry how proline substitutions, commonly used for SARS-CoV-2 spike stabilisation in vaccine design, directly affects ACE2 receptor interactions *via* dynamics of open and closed states.

## Graphical abstract

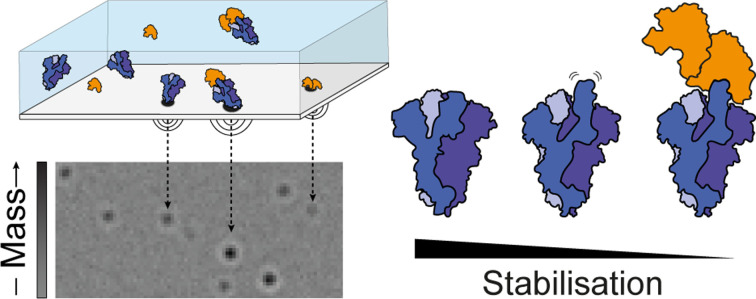

The SARS-CoV-2 spike (S) is a class I fusion glycoprotein complex responsible for host cell entry and is the basis for current COVID-19 vaccines. The spike is a metastable trimer, consisting of S1 and S2 heterodimers, and the introduction of two proline substitutions (K986P, V987P) to the S2 central helix significantly stabilises S in its prefusion state.^1^ These so-called “2P” constructs enabled determination of a high-resolution structure *via* cryoEM (Fig. 1a).^1^ Importantly, the majority of approved COVID-19 vaccines exploit 2P stabilisation for optimal antigenicity of expressed immunogens. More recent work has led to the generation of a further stabilised spike termed “HexaPro” (K986P, V987P, F817P, A892P, A899P and A942P) which improved expression yields ∼10 fold compared to 2P and are attractive candidates for next-generation vaccines.^2^ Here, using mass photometry (MP) we reveal that 2P and HexaPro spikes are functionally dissimilar in their capability to bind the host receptor angiotensin-converting enzyme-2 (ACE2) and is attributed to receptor binding domain (RBD) dynamics among wild-type Wuhan and Omicron SARS-CoV-2 strains.

**Fig. 1:**
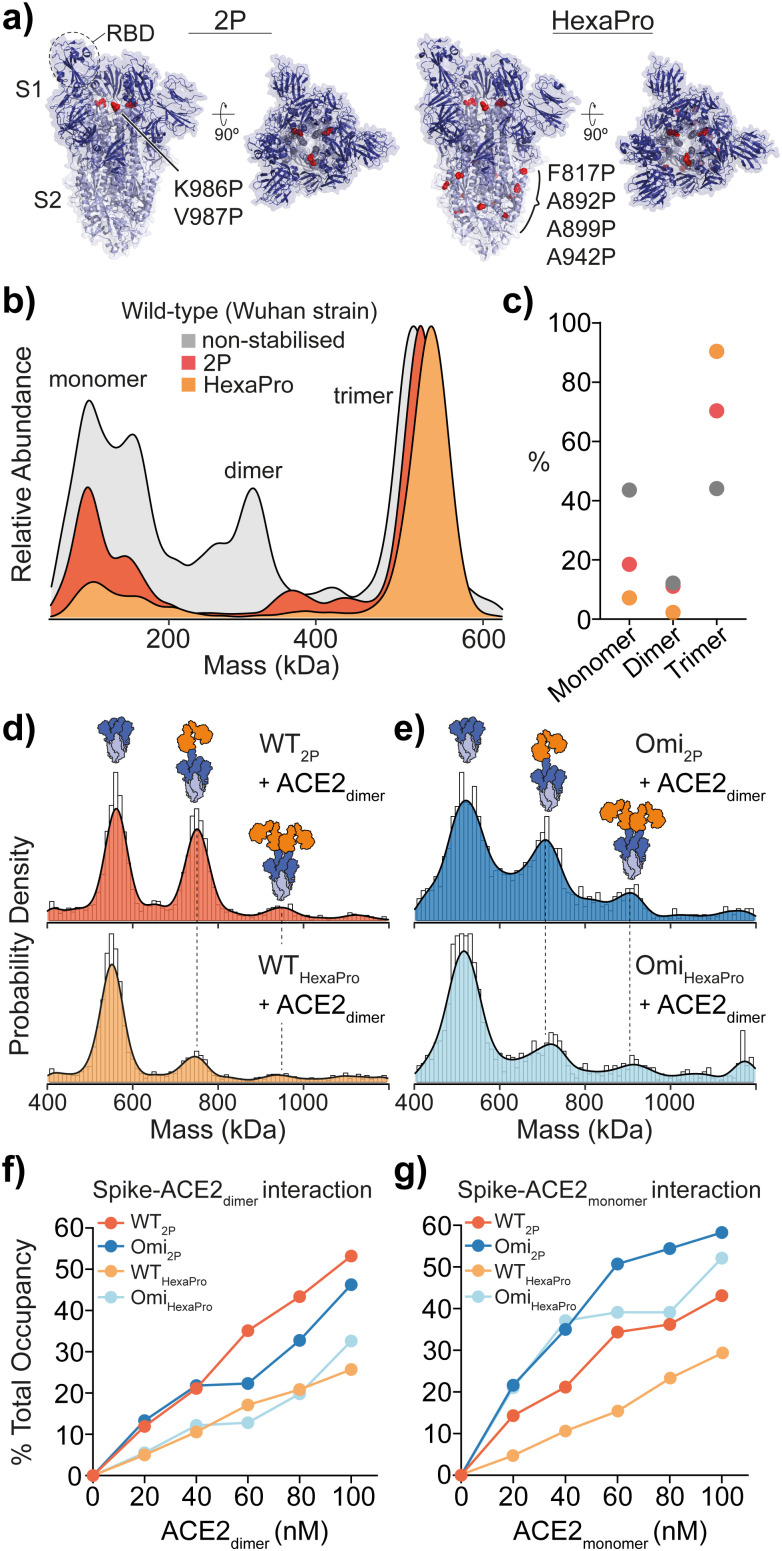
Structure and mass photometry (MP) of spike with ACE2 binding. (a) The structures of 2P and HexaPro spike with one receptor binding domain (RBD) in the up position (2P: PDB ID: 6VSB, HexaPro: PDB ID: 6XKL). Proline mutations are highlighted in red and S1 and S2 domains are dark and light blue respectively. (b) Overlaid MP kernel density plot of wild-type (Wuhan) non-stabilised, 2P and HexaPro spikes, with the percentage contribution of monomer, dimer and trimer species (c). MP of spike-ACE2 interactions of (d) WT 2P and HexaPro with dimeric ACE2 and (e) Omicron 2P and HexaPro with dimeric ACE2 (4 : 1 ratio of ACE2 to spike is shown). MP of spike-ACE2 binding of (f) dimeric and (g) monomeric ACE2 across a range of ACE2 concentrations (the extent of binding is plotted as a percentage total occupancy).

MP is a single molecule imaging method that reports the mass distribution of non-labelled proteins *via* interferometric light scattering.^3^ MP is highly sensitive and intrinsically quantitative in measuring protein complexes based on counting individual species. We have recently demonstrated the distinctive advantage of MP for quantifying protein–protein interactions *via* molecular counting over ensemble-based biophysical methods, including surface plasmon resonance (SPR).^3^

MP analysis of soluble recombinant 2P and HexaPro spike protein (Wuhan-hu-1 strain, herein referred to as wild-type) as well as the non-stabilised equivalent enabled the identification of varying distributions of monomeric, dimeric and trimeric spike species. A distinct decrease in spike monomers and dimers as a function of proline substitutions was observed (Fig. 1b and c). Not surprisingly, HexaPro was the most stable and was predominantly trimeric (90% of total spike molecule counts, 550 kDa) while non-stabilised trimers dissociated into smaller subunits (Table S1, ESI†). Next, we explored binding of 2P and HexaPro trimers to ACE2 in either its monomeric or dimeric form. MP confirmed ACE2 proteins expressed without the neck domain were 100% monomeric while ACE2 with the neck domain existed as both monomers and dimers, which is a result of the sample concentration (10 nM) being near the monomer-dimer dissociation constant (*K*_d_) during the MP measurement (Fig. S1, ESI†). Mixing spike and dimeric ACE2 at a 1 : 4 ratio showed wild-type (WT) 2P bound one and two receptors at 42% and 8% respectively (Fig. 1d, top). WT HexaPro bound fewer, with 19% 1 ACE2-spike and 7% 2 ACE2-spike complexes (Fig. 1d, bottom). This was consistent for Omicron 2P and HexaPro spikes (B.1.1.529 strain) under the same conditions (Fig. 1e). This highlights a clear discrepancy in the current biophysical understanding of spike-ACE2 binding. SPR data of ACE2 binding to WT 2P and HexaPro spikes reported equivalent *K*_d_ values (11.3 nM (2P) and 13.3 nM (HexaPro)).^2^ In order to rationalise the equal affinities observed by SPR in light of our MP data that clearly shows reduced ACE2 binding with HexaPro, and considering the fact that RBD amino acid sequences are the same, we hypothesised we are observing altered conformational dynamics, with 2P spikes adopting structures with more RBDs in the ACE2-accesible “up” state. The occurrence of RBD dynamics is well known but quantifying spike conformational states and the number of ACE2-bound RBDs is largely based on cryoEM studies, which may not accurately quantify these states. It is essential to point out that cryo-EM studies have either been completed using spike trimers plus ACE2 monomers^4,5^ or using S1 subunits plus ACE2 dimers.^6^

Measuring the percentage of bound ACE2 (*i.e.* total occupancy, calculated using the sum of spike counts including species with 0, 1, 2 and 3 ACE2 molecules bound and expressed as a percentage of total) from 0 to 100 nM with spike kept constant at 25 nM, showed a greater occupancy of 2P spikes compared with HexaPro (Fig. 1f and Fig. S2 and Table S2, ESI†). WT and Omicron HexaPro spikes were less occupied but behaved similar across dimeric ACE2 concentrations. The percent occupancy for Omicron-ACE2 monomer was greater compared to WT among ACE2 concentrations tested (Fig. 1g and Fig. S3 and Table S3, ESI†). On average, the percent total occupancy was more for monomer ACE2 compared to dimer ACE2 at the same receptor concentrations. Monomeric ACE2 binding was different, specifically in that we observed up to three equivalents bound with Omicron 2P and HexaPro at 80 and 100 nM ACE2 (3.2 and 4 equivalents of ACE2) (Fig. S4, ESI†). This also indicates that structural architecture influences RBD binding properties and dimeric ACE2, the physiologically relevant oligomeric form, encounters more steric hindrance in spike engagement. Unlike ACE2 monomers, spikes with three ACE2 dimers bound were not detected, even at 4 : 1 ACE2-spike ratios. To establish that the observed differences in binding was ACE2 specific and not due to a global change in protein architecture or (un)folding, we used two antibodies that target the RBD (IgG clone EY6A) and N-terminal domain (NTD, IgG clone 288)^8^ that were isolated from COVID patients (Fig. S5, ESI†). Importantly, spike binding with the RBD antibody was consistent with ACE2 MP data, confirming the presence of varying dynamics between 2P and HexaPro (Fig. S5a and b, ESI†). Expectedly, NTD binding was equivalent for 2P and HexaPro spikes as these domains should not undergo dynamical shifts (Fig. S5c and d, ESI†).

The SARS-CoV-2 spike is heavily glycosylated and site-specific glycosylation has been proposed to modulate ACE2 binding.^7,9^ We speculated that differences in glycan processing may contribute to variations in ACE2 binding we observe. To test this, we first profiled global differences in glycosylation *via* ultra-high performance liquid chromatography (UHPLC), which provides a quantitative readout of released and fluorescently labelled *N*-glycans. To simplify quantitation, we compared the fold-change for *N*-glycans *via* their UHPLC peak area following sialic acid removal (Fig. 2a). Although minor differences were apparent when comparing individual peaks between 2P and HexaPro spikes, these changes were not fully consistent across strains, suggesting stabilisation does not significantly affect glycan processing (Fig. 2a and TableS4 and S5, ESI†). UHPLC profiling following an additional Endoglycosidase H digestion, which cleaves oligomannose and hybrid type structures, yielded nearly superimposable chromatograms (Fig. S6, ESI†) indicating equivalent glycosylation between spikes.

**Fig. 2:**
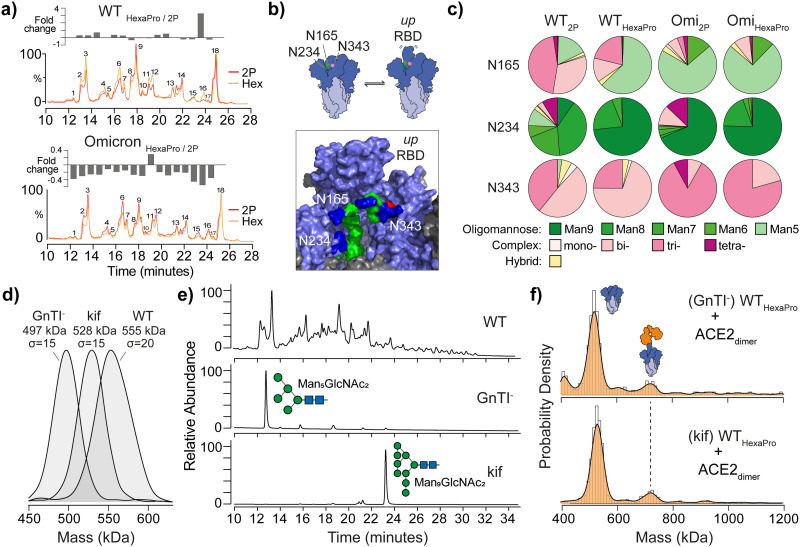
The effect of spike glycan processing on ACE2 binding. (a) Overlaid UHPLC chromatograms of de-sialylated *N*-glycans from WT 2P and HexaPro (top) and Omicron spikes (bottom). Plot of fold-changes for each of the 18 most abundant integrated glycan peak areas is shown above each chromatogram. (b) Schematic representation of glycan gates at positions N165, N234 and N343 (image created from Casalino *et al*.^7^ spike model and PDB ID: 6VSB). Glycans shown are Man_5_GlcNac_2_ (N165), Man_9_GlcNac_2_ (N234) and G0F, a core-fucosylated bi-antennary complex-type structure, (N343); mannose (green), *N*-acetylglucosamine (blue) and fucose (red). (c) Pie charts reflecting site-specific glycosylation analysis of N165, N234 and N343. Complex-type glycans are classified by mono-, bi-, tri- and tetra-antennary structures. (d) MP of WT HexaPro spike and spikes with homogenous glycosylation produced in GnTI- deficient cells or in the presence of kifunensine (kif). (e) UHPLC of *N*-glycans from WT, GnTI^−^ and kif derived HexaPro spikes confirming the presence of Man_5_GlcNAc_2_ (GnTI^−^) and Man_9_GlcNAc_2_ (kif) structures. (f) MP of GnTI^−^ and kif wild-type (WT, Wuhan) HexaPro spikes with dimeric ACE2 (4 : 1 ratio of ACE2 to spike is shown).

Specific spike *N*-glycans at N165, N234 and N343 have been suggested to play a modulating role in RBD “up” positioning and ACE2 binding (Fig. 2b).^7,9^ To explore if structural differences at these critical sites may explain 2P and HexaPro spike-ACE2 binding, we identified site-specific glycans *via* glycoproteomics (Fig. 2c). All sites were fully occupied and varied by glycan class (*i.e.* oligomannose, complex or hybrid type), with subtle differences in structure at each site. N343 has been dubbed a “glycan gate”^11^ and we identified these glycans as predominantly complex-type across all samples. N165 exhibited some heterogeneity with WT having more bi- and tri-antennary complex structures (Table S6 and S7, ESI†). Omicron contained more oligomannose structures, specifically Man_5_GlcNAc_2_, glycans (Table S8 and S9, ESI†). N234 is interesting in that Man_9_GlcNAc_2_ (an unprocessed oligomannose glycan) has been indicated *via* molecular dynamics to stabilise RBDs in the “down” position *via* hydrogen bond networking to neighbouring amino acids. We observed an increase in Man_9_GlcNAc_2_ on WT HexaPro compared to WT 2P, which may explain changes in ACE2 binding *via* RBD stabilisation, however this increase was less profound with Omicron samples.

To establish the degree to which glycosylation at N234, and other sites, modulates RBD dynamics and differences in ACE2 binding, we generated spikes with homogenous and defined *N*-glycan structures. Specifically, Man_5_GlcNAc_2_ containing spikes were produced in *N*-acetylglucosaminyltransferase I (GnTI^−^) deficient cells and Man_9_GlcNAc_2_ containing spikes were expressed using HEK 293F cells in the presence of kifunensine (kif), a potent endoplasmic reticulum (ER) α-mannosidase inhibitor. The expected mass differences between glycoengineered spikes was evident by MP, while also highlighting the glycan heterogeneity of WT spike by the broader mass distribution (*σ* = 20 compared to *σ* = 15 for each glycoengineered spike, Fig. 2d). The presence of Man_5_GlcNAc_2_ and Man_9_GlcNAc_2_*N*-glycans was confirmed using UHPLC (Fig. 2e), but no noticeable difference in ACE2 binding was seen with either GnTI^−^ or kif-produced WT HexaPro spikes (Fig. 2f). The greater binding capacity of 2P spikes to ACE2 over HexaPro, regardless of GnTI^−^ or kif origin, was also observed and consistent with naturally glycosylated trimers (Fig. S7, ESI†). This shows that changes in glycan structure at N165, N234 or N343 have no obvious effect in contributing to RBD positioning and differences in ACE2 binding as a consequence of stabilisation.

Hydrogen–deuterium exchange (HDX) mass spectrometry has shown spikes can readily adopt open trimer conformations, which was proposed to enable greater accessibility of the RBD to ACE2.^10^ The open state was found to be temperature dependent and favoured at 4 °C. It was also observed that 2P spikes had a greater propensity to adopt an open conformation than HexaPro.^10^ Even though ACE2 binding was not explored, the HDX-MS data are valuable and allow us to test whether the differences in ACE2 binding we see are due to dynamics or potentially other causes.

Spikes preincubated at 37 °C should in principle adopt a closed structure and subsequently bind ACE2 to a lesser extent. We detected a clear reduction in ACE2 binding after a 1 hour 37 °C preincubation across WT spikes (Fig. 3a, b and Fig. S8, ESI†) with both dimeric and monomeric ACE2 (Fig. 3c, d and Fig. S9 and Table S10, ESI†). Interestingly, the reduction in binding with HexaPro preincubated at 37 °C was less than 2P (0.7 *vs.* 1.0 mean fold change) (Fig. 3c, d and Table S10, ESI†). Although preincubation of all spike proteins at 37 °C reduced ACE2 binding, the capacity of 2P spikes was greater than HexaPro (26% *vs.* 15% mean occupancy (Table S10, ESI†), suggesting that a subpopulation of 2P spike remains in an ACE2-accessible conformation independent of temperature. Our MP values were consistent with HDX-MS data, at 4 °C and 37 °C the proportion of spikes in the open state was greater for 2P compared to HexaPro (40% *vs.* 9% post 37 °C incubation at the maximum time points measured).^10^ Our data expands these observations in the context of ACE2 binding and provides quantitative functional relevance for temperature-dependent states of 2P and HexaPro stabilised spikes.

**Fig. 3:**
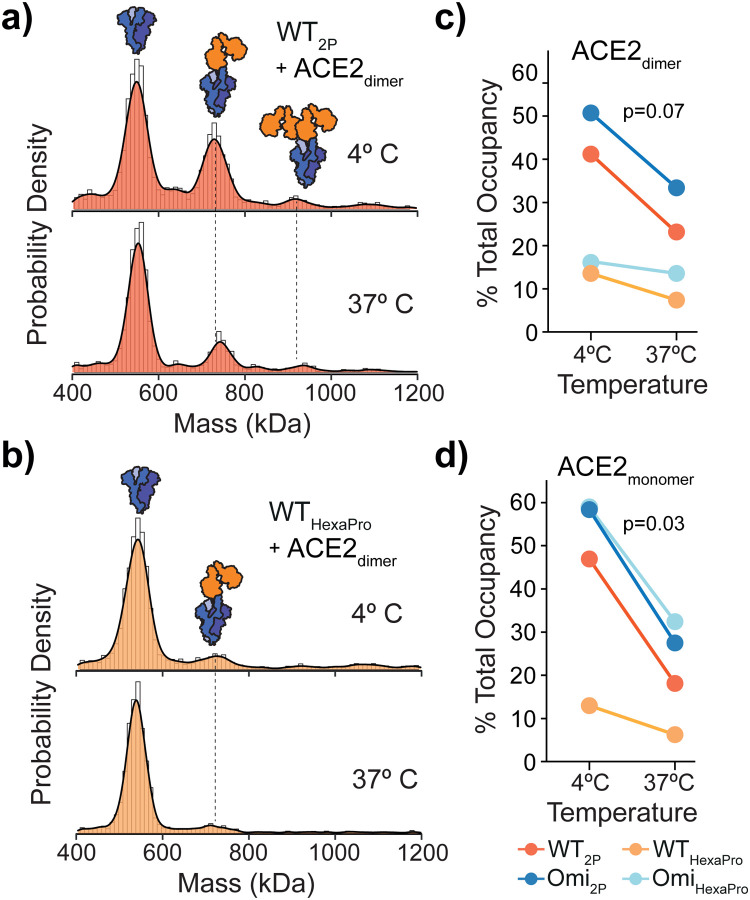
Temperature effects on spike dynamics and ACE2 binding. MP of spike-ACE2 binding following spike incubation on ice or at 37 °C prior to the addition of monomeric (a) or dimeric (b) ACE2 (4 : 1 ratio of ACE2 to spike is shown). The extent of ACE2 binding was represented as percentage total occupancy relative to total MP (protein) counts at both temperatures for WT and Omicron 2P and HexaPro spikes with ACE2 dimer (c) or monomer (d). *P*-values were determined by a paired Student's *t*-test.

In conclusion, we reveal how proline stabilisation of the SARS-CoV-2 spike directly alters ACE2 binding stoichiometry for both Wuhan-hu-1 and Omicron-B.1.1.529 strains. Differences in ACE2 binding were not driven by changes in glycan processing and therefore RBD dynamics *via* glycan gating may depend solely on the presence/absence of glycans (macroheterogeneity) at these sites rather than changes in structure (microheterogeneity). It is worthy to note these *N*-glycosylation sites are preserved among SARS-CoV-2 variants to date.

Our data quantitatively shows that 2P spikes sample open conformations to a greater extent than HexaPro resulting in an increase in ACE2 binding *via* greater RBD accessibility. Further evidence for 2P spike shifts towards the open conformation was confirmed by antibody binding to the RBD and a non-neutralizing epitope on S2 that is more accessible with 2P than HexaPro.^11^ Importantly, the use of HexaPro was shown to be superior over 2P and non-stabilised spike vaccines through inducing a more potent neutralisation response in preclinical models.^12^ The HexaPro vaccine candidate (NDV-HXP-S) also induced a higher ratio of neutralising compared to non-neutralising antibodies *versus* 2P encoding mRNA vaccines in humans.^13^ It could be envisaged that the increase in expression gained by the additional proline substitutions in HexaPro, alongside the altered repertoire of antibodies generated as a result of immunisation, will provide added benefit over current vaccines. However, researchers using stabilised spikes to study function or structure should consider that these alterations may not represent native spike dynamics found on the surface of the virus, as evidenced through altered ACE2 binding in our study.

This work was supported by a UKRI Future Leaders Fellowship [MR/V02213X/1]. W. B. S. and S. A. B. acknowledge BBSRC/UKRI BB/V011456/1 funding and support of the philanthropic donors to the University of Oxford's COVID-19 Research Response Fund. The authors thank Dr Juthathip Mongkolsapaya, Prof. Gavin Screaton, Lachlan Deimel and Prof. Quentin Sattentau (University of Oxford) for the antibodies. We also thank Dr Roi Asor and Prof. Justin Benesch for valuable discussions.

## Conflicts of interest

W. B. S. is a shareholder and consultant to Refeyn Ltd. S. A. B. declares no conflict of interest.

## Supplementary Material

CC-058-D2CC04711J-s001
