# Supplementary material for: Mass photometry reveals SARS-CoV-2 spike stabilisation to impede ACE2 binding through altered conformational dynamics
Source: Chem Commun (Camb). 2022 Oct 28;58(93):12939–42. doi: 10.1039/d2cc04711j (PMC9680610; doi:10.1039/d2cc04711j)
Supplement: CC-058-D2CC04711J-s001 [file CC-058-D2CC04711J-s001.pdf]

Electronic Supplementary Material (ESI) for ChemComm.
This journal is © The Royal Society of Chemistry 2022

SUPPORTING INFORMATION

Mass photometry reveals SARS-CoV-2 spike stabilisation to impede ACE2 binding through altered conformational dynamics

Sean A. Burnap[a, b] and Weston B. Struwe[*a, b]

[a] Physical and Theoretical Chemistry Laboratory, Department of Chemistry, University of Oxford, South Parks Road, OX1 3TA.

[b] The Kavli Institute for Nanoscience Discovery, Dorothy Crowfoot Hodgkin Building, South Parks Road, OX1 3QU.

*Correspondence to: weston.struwe@chem.ox.ac.uk

Contents

1. Materials and Methods 3
1.1 Protein expression constructs 3
1.2 Protein expression and purification 3
1.3 Mass photometry 3
1.4 Glycan UHPLC 4
1.5 Glycoproteomics 4
2. Data Availability 5
3. Supplementary Figures 6
Figure S1. Mass photometry of ACE2 monomer and dimer samples 6
Figure S2. ACE2 dimer titration MP of 2P and HexaPro spike 7
Figure S3. ACE2 monomer titration MP of 2P and HexaPro spike. 8
Figure S4. Percentage bound monomeric ACE2 to WT and Omicron 2P and HexaPro spikes 9
Figure S5. MP of WT 2P and HexaPro with NTD and RDB targeting antibody 10
Figure S6. N-glycan UHPLC chromatograms 11
Figure S7. MP of glycoengineered spikes with dimeric ACE2. 12
Figure S8. Dimeric ACE2 binding to temperature treated spikes. 13
Figure S9. Monomeric ACE2 binding to temperature treated spikes. 14
4. Supplementary Tables 15
Table S1. MP of non-stabilised, 2P and HexaPro Wuhan spike. 15
Table S2. ACE2 dimer titration MP of 2P and HexaPro spike 15
Table S3. ACE2 monomer titration MP of 2P and HexaPro spike 15
Table S4. WT N-glycan UHPLC quantitation 16
Table S5. Omicron N-glycan UHPLC quantitation 17
Table S6. WT 2P site-specific glycan quantification 18
Table S7. WT HexaPro site-specific glycan quantification 18
Table S8. Omicron 2P site-specific glycan quantification 19
Table S9. Omicron HexaPro site-specific glycan quantification 19
Table S10. The effect of temperature upon spike ACE2 binding 20
5. References 21

1. Materials and Methods

1.1 Protein expression constructs

Non-stabilised SARS-CoV-2 spike expression plasmid (pαH-S-GSAS, Ectodomain a.a's 1-1208, 682-685 furin site = RRAR replaced with GSAS) was from Addgene (plasmid # 164565, deposited by P. Acharya).[1] The vector pCAGGS encoding the Wuhan-hu-1 2P spike trimer was a kind gift from the Krammer Laboratory, Department of Microbiology, Icahn School of Medicine at Mount Sinai, New York.[2] Wuhan-hu-1 HexaPro spike expression construct was a gift from the McLellan Laboratory, Department of Molecular Biosciences, The Univeristy of Texas, Austin (Addgene plasmid # 154754).[3] The pHL-sec vector encoding Omicron 2P spike was a kind gift from the Townsend Laboratory, Weatherall Institute of Molecular Medicine, University of Oxford. The pHL-sec vector encoding Omicron HexaPro was a kind gift from the Stuart Laboratory, Division of Structural Biology, University of Oxford. pHL-sec vectors encoding monomeric (a.a 19-611) and dimeric (a.a 19-726) ACE2 were a kind gift from the Zitzmann Laboratory, Department of Biochemistry, University of Oxford.

1.2 Protein expression and purification

Proteins were transiently expressed in HEK293F (FreeStyle™, Thermo Fisher Scientific) or HEK293S GnTI^{-} (ATCC, CRL-3022) cells. Cells were cultured in Freestyle 293 expression media (ThermoFisher Scientific) and incubated at 37 °C, 8% CO2 and 120 rpm. Transfection was achieved using FreeStyle™ MAX reagent (Invitrogen) and OptiMEM™ (Gibco) following a published protocol.[4] Kifunensine was added at time of transfection at a final concentration 10 µM. Five days post transfection, cell culture supernatant was harvested by centrifugation at 3000 x g for 10 min and then filtered using 0.45 µM pore size filters (Merck). Supernatants were supplemented with 10 mM imidazole and His-tagged spikes were purified using a HisTrap HP, 5mL column (Cytiva) connected to an ÄKTA pure protein purification system (Cytiva). Proteins were further purified by size exclusion chromatography (SEC) using a Superose 6 increase 10/300 GL column (GE Healthcare) equilibrated in Dulbecco's phosphate-buffered saline (DPBS, pH 7.4, ThermoFisher Scientific). Trimer containing SEC fractions were pooled and concentrated using Amicon molecular weight cut-off centrifugal filters (GE Healthcare). Protein concentrations were determined using a Nanodrop spectrophotometer (Thermo Fisher Scientific) at absorbance 280 nM and corrected for protein molecular weight and extinction coefficient.

1.3 Mass photometry

Mass photometry measurements were conducted using a Refeyn TwoMP system (Refeyn Ltd) as previously described.[5] High Precision No. 1.5H glass coverslips were cleaned via sonication in Milli-Q H_2O, followed by isopropanol and Milli-Q H_20 then dried under nitrogen flow. Sample chambers were assembled using silicone gaskets (CultureWell™ reusable gasket, 3mm diameter x 1 mm depth, Grace Bio-Labs). Coverslips were placed on the MP sample stage and a single gasket was filled with 5-20 µL DPBS (without calcium, without magnesium, pH 7.4 ThermoFisher Scientific) to find focus and ensure low background signal-to-noise. Spike samples were measured at a final concentration of 10 nM. For ACE2 interaction experiments, 25 nM spike was added to 0 to 100 nM ACE2 and equilibrated for 5 minutes prior to data acquisition.

Acquisition settings within AcquireMP (v2.5.0, Refeyn Ltd) were as follows: regular field of view, frame binning = 2, frame rate = 498.3 Hz, pixel binning = 6, exposure time 1.95 ms and movies were taken over 60 seconds. Mass calibration was conducted using an in-house protein standard. Data was analysed using DiscoverMP (v2.5.0, Refeyn Ltd). Molecule counts

were used to determine levels of spike-ACE2 occupancy. The interaction between spike and ACE2 is represented as % total occupancy, which was calculated using the sum of all spike counts including species with 0, 1, 2 and 3 ACE2 molecules bound and expressed as a percentage of 1, 2 and 3 ACE2 bound counts compared to total spike counts. Representative histograms with overlaid kernel density estimates were generated in R (v4.2.1) using event exports from DiscoverMP.

1.4 Glycan UHPLC

Approximately 10 µg spike was loaded onto SDS-PAGE gels, run, excised and de-stained. PNGase F (generated in-house) was added to each gel-band and incubated for 16 hours at 37°C. Released N-glycans were labelled with 2-aminoanthranilic acid (2-AA) as previously described.[6] Briefly, glycans were resuspended in 30 µL of HPLC-grade H_2O followed by addition of 80 µL of labelling mixture (30 mg/mL 2-AA and 45 mg/mL sodium cyanoborohydride in a solution of sodium acetate trihydrate [4% w/v] and boric acid [2% w/v] in methanol). N-glycans were incubated at 80 °C for 1 hour. Excess label was removed using Spe-ed Amide-2 cartridges (Applied Separation) as described.[6]

Fluorescently labelled N-glycans were profiled by hydrophilic interaction liquid chromatography-ultra high performance liquid chromatography (HILIC-UHPLC) using a 2.1 mm × 10 mm Acquity BEH Amide Column (1.7 µm particle size) (Waters, Elstree, UK). The mobile phase was solvent A: 50 mM ammonium formate, pH 4.4 and solvent B: MeCN. The gradient was: (t = 0): 22.0% A, 78.0% B (flow rate of 0.5 mL/min); t = 38.5: 44.1% A, 55.9% B (0.5 mL/min); t = 39.5: 100% A, 0% B (0.25 mL/min); t = 44.5: 100% A, 0% B (0.25 mL/min); t = 46.5: 22.0% A, 78.0% B (0.5 ml/min), t = 48: 22.0% A, 78.0% B (0.5 mL/min). Fluorescence was measured using an excitation wavelength of 360 nm and a detection wavelength of 425 nm.

Endoglycosidase H (New England Biolabs) was added to 2-AA labelled glycans for 16 hours at 37 °C, according to manufacturer's instructions. Digested glycans were purified using a PVDF protein-binding membrane plate (Millipore) prior to HILIC-UHPLC analysis. The same procedure was conducted for sialic acid removal using α2-3,6,8 neuraminidase (New England Biolabs). Data processing was performed using Empower 3 software (Waters, Elstree, UK). Glycan peaks were integrated within Empower 3 and resulting peak areas were exported. Peak areas were normalised to the N-glycan peak labelled 18 across each spike preparation and plotted as relative percentages. Fold changes of aligned peak areas were also calculated between 2P and HexaPro spikes across both WT and Omicron strains.

1.5 Glycoproteomics

Approximately 5 µg protein was loaded and run on an SDS-PAGE. Gel bands were excised and washed sequentially with HPLC grade water followed by 1:1 (v/v) $MeCN/H_2O$. Gel bands were dried (via vacuum centrifuge), treated with 10 mM dithiothreitol (DTT) in 100mM NH_4HCO_3 and incubated for 45 minutes at 56°C with shaking. DTT was removed and 55 mM iodoacetamide (in 100 mM NH_4HCO_3) was added and incubated for 30 minutes in the dark. All liquid was removed and gels were washed with 100 mM NH_4HCO_3/MeCN as above. Gels were dried and 12.5 ng/µl trypsin, chymotrypsin or alpha lytic protease was added separately and incubated overnight at 37°C. Samples were then washed and (glyco)peptides were extracted and pooled with sequential washes with 5% (v/v) formic acid (FA) in H_2O and MeCN. Dried samples were reconstituted in 2% MeCN, 0.05% trifluoroacetic acid and run by LC-MS.

Samples were analysed using an Ultimate 3000 UHPLC coupled to an Orbitrap Eclipse Tribrid mass spectrometer (Thermo Fisher Scientific). Peptides were loaded onto a 75 µm × 2 cm pre-

column and separated on a 75 µm × 15 cm Pepmap C18 analytical column (Thermo Fisher Scientific). Buffer A was 0.1% FA in H_2O and buffer B was 0.1% FA in 80% MeCN with 20% H_2O. A 40 minute linear gradient (0% to 40% buffer B) was used. To maximise glycopeptide identification two MS acquisition methods were utilised. First, a universal HCD identification method was used. Data was collected in data-dependent acquisition mode with a mass range 300 to 2000 m/z and at a resolution of 120000. For MS/MS scans, HCD normalized energy was set to 30% with orbitrap detection at a resolution of 30000. Secondly, a triggered EThcD method was used. Data was collected in data-dependent acquisition mode with a mass range 350 to 1800 m/z and at a resolution of 120000 including charge states 2-8. Triggering MS HCD scans, collision energy mode was set to fixed at a collision energy of 28% and orbitrap resolution 30000. EThcD was triggered upon the detection of glycopeptide-specific oxonium ions (HexNac: 204.0867 m/z, HexNac fragment: 138.0545 m/z and HexNacHex: 366.1396 m/z) with a 15 ppm mass tolerance, and data was collected using the orbitrap at a resolution of 30000.

Glycopeptide data was analysed with Byonic (Protein Metrics). Digestion was set to RK, TASV and FYWML for trypsin, alpha-lytic and chymotrypsin digests, respectively and fully specific with a maximum of two miss cleavages allowed. Carbamidomethylation (57.02 Da) was set as a fixed modification, while methionine oxidation (15.99 Da), deamidation (0.98 Da) and Gln -> pyro-glutamate (-17.03 Da) were set as variable modifications. The Byonic in-built common human N-linked (132 glycans) and O-linked glycan (9 glycans) databases were used to identify glycopeptides.

Byonic output files were imported into Byologic for quantification (Protein Metrics). A minimum Byonic threshold score of 100 was used for glycopeptide identification. All glycopeptide assignments were manually validated. For quantification, the extracted ion chromatogram intensities for each glycopeptide and unoccupied peptides were summed and plotted relative to the total intensity for each glycosite.

2. Data Availability

Mass spectrometry raw data have been deposited to the ProteomeXchange Consortium (http://proteomecentral.proteomexchange.org) via the PRIDE partner repository with the dataset identifier <PXD>.[7]

3. Supplementary Figures

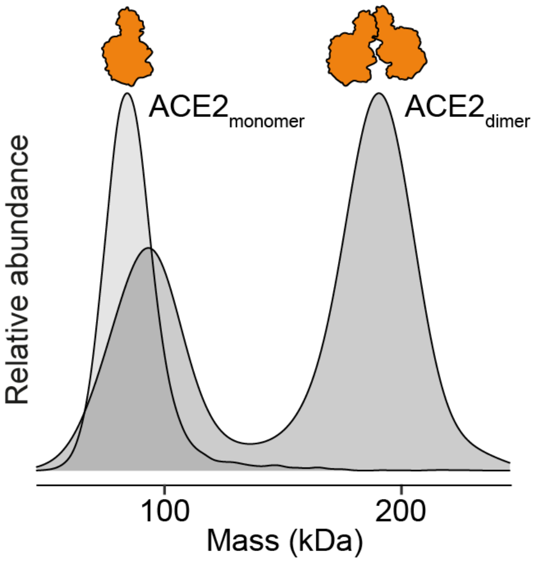

Figure S1. Mass photometry of ACE2 monomer and dimer samples. ACE2 monomers (a.a 19-611) yielded a single peak at 80 kDa (light grey). ACE2 dimers (a.a 19-726) were present in both dimeric and monomeric forms at 96 kDa and 189 kDa respectively (dark grey). Each sample was measured at 10 nM final concentration.

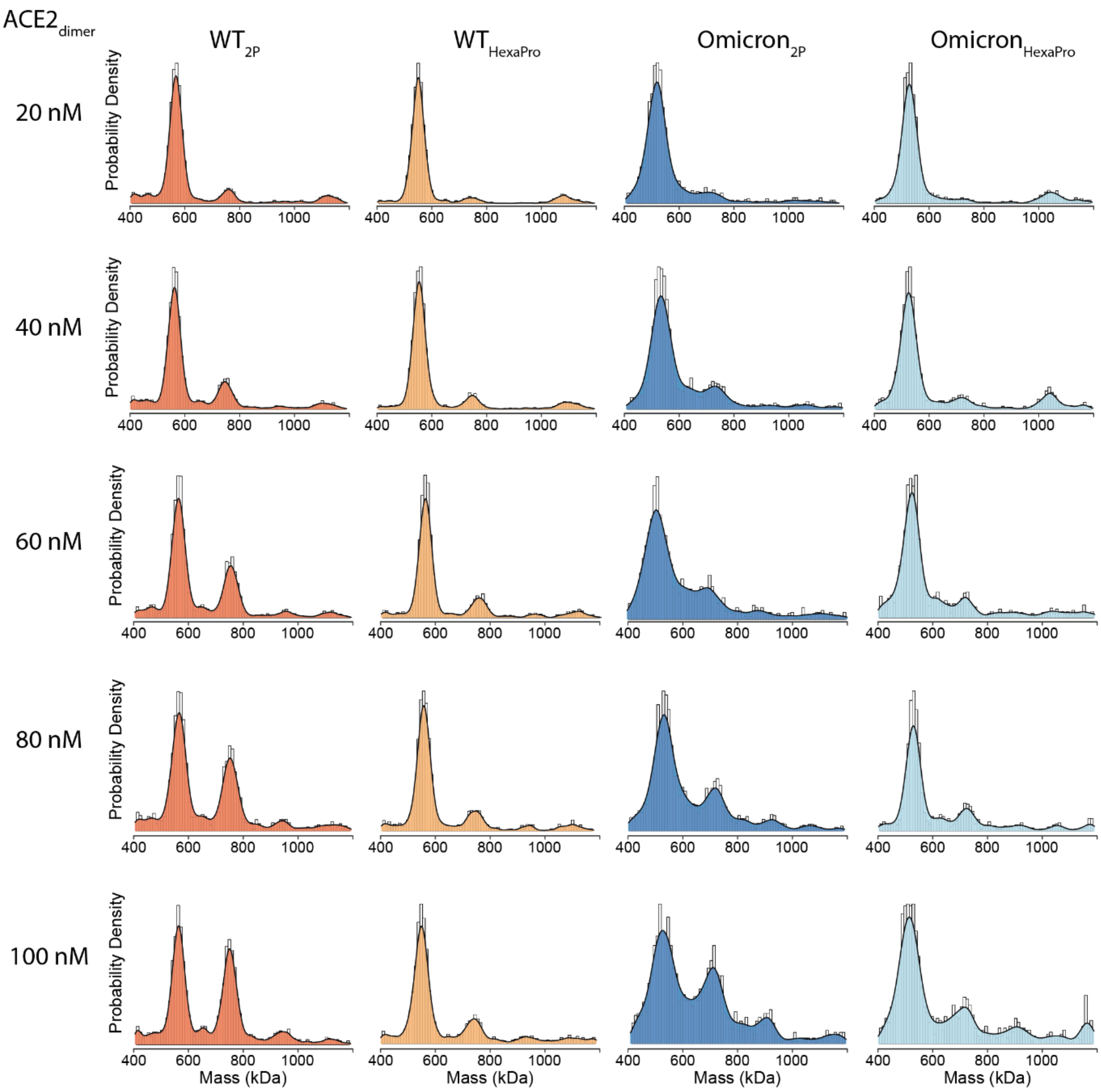

Figure S2. ACE2 dimer titration MP of 2P and HexaPro spike. Interaction measured between 2P and HexaPro spikes (WT and Omicron) with 20-100 nM dimeric ACE2. Representative histograms with overlaid kernel density estimates are shown. The concentration of spike was kept constant at 25 nM and measurements were taken 5 minutes after mixing of spike and ACE2 at room temperature.

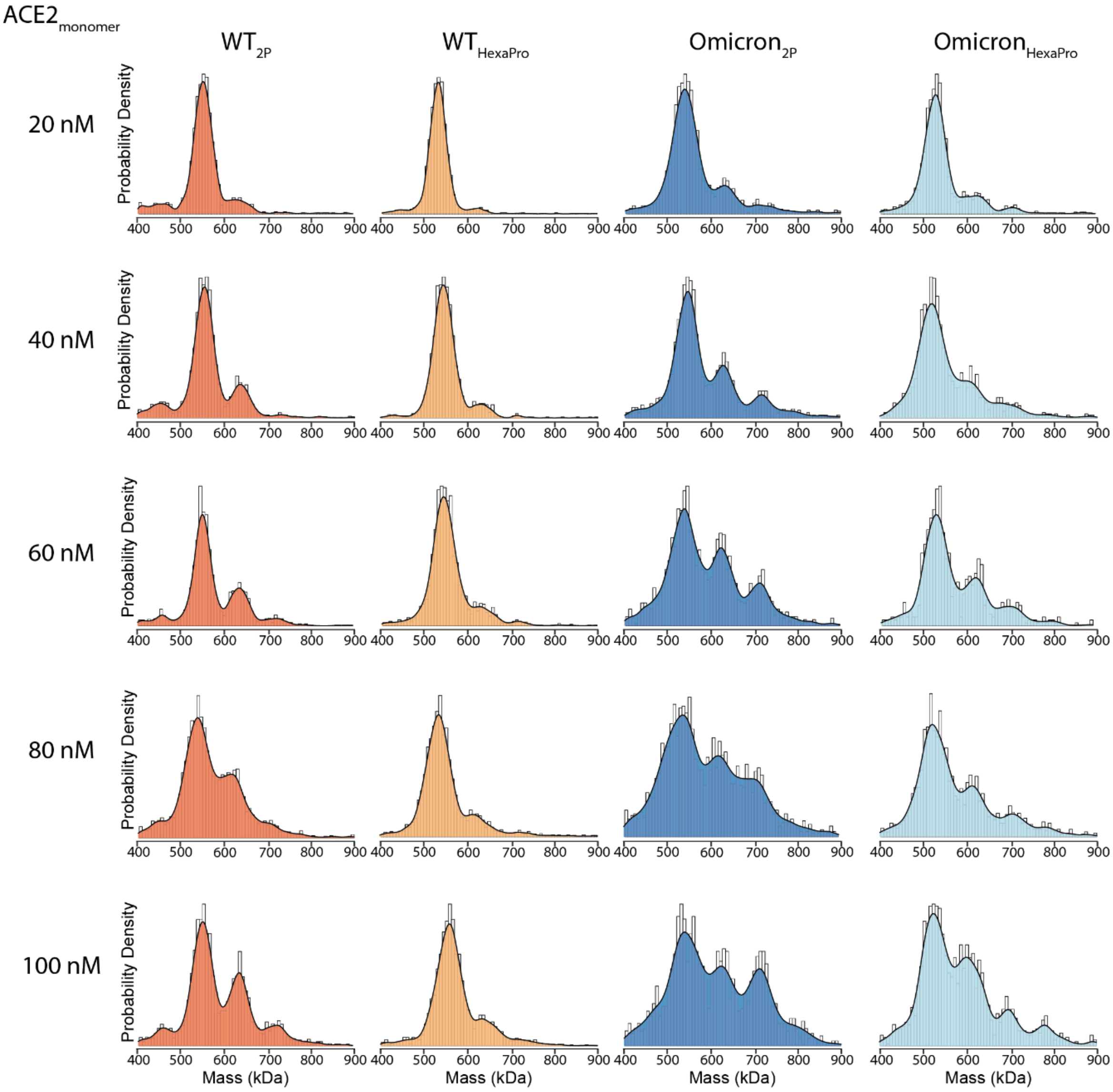

Figure S3. ACE2 monomer titration MP of 2P and HexaPro spike. Interaction measured between 2P and HexaPro spikes (WT and Omicron) with 20-100 nM monomeric ACE2. Representative histograms with overlaid kernel density estimates are shown. The concentration of spike was kept constant at 25 nM and measurements were taken 5 minutes after mixing of spike and ACE2 at room temperature.

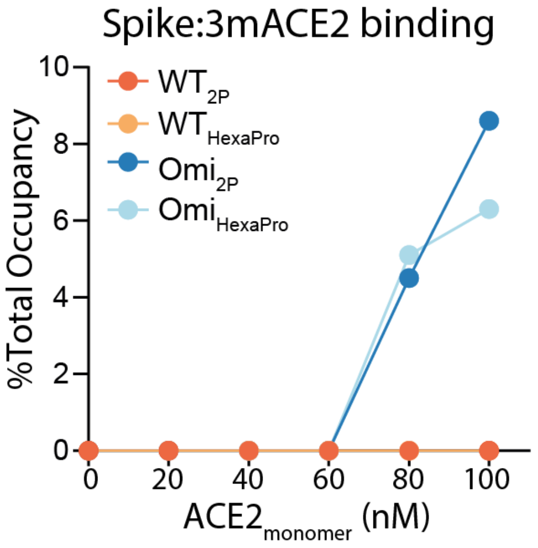

Figure S4. Percentage bound monomeric ACE2 to WT and Omicron 2P and HexaPro spikes. The extent of spike binding to 3 monomeric ACE2 proteins was plotted as a percentage of total occupancy compared to total spike counts. Omicron spike bound up to 3 ACE2 monomers which was not observed for WT spike.

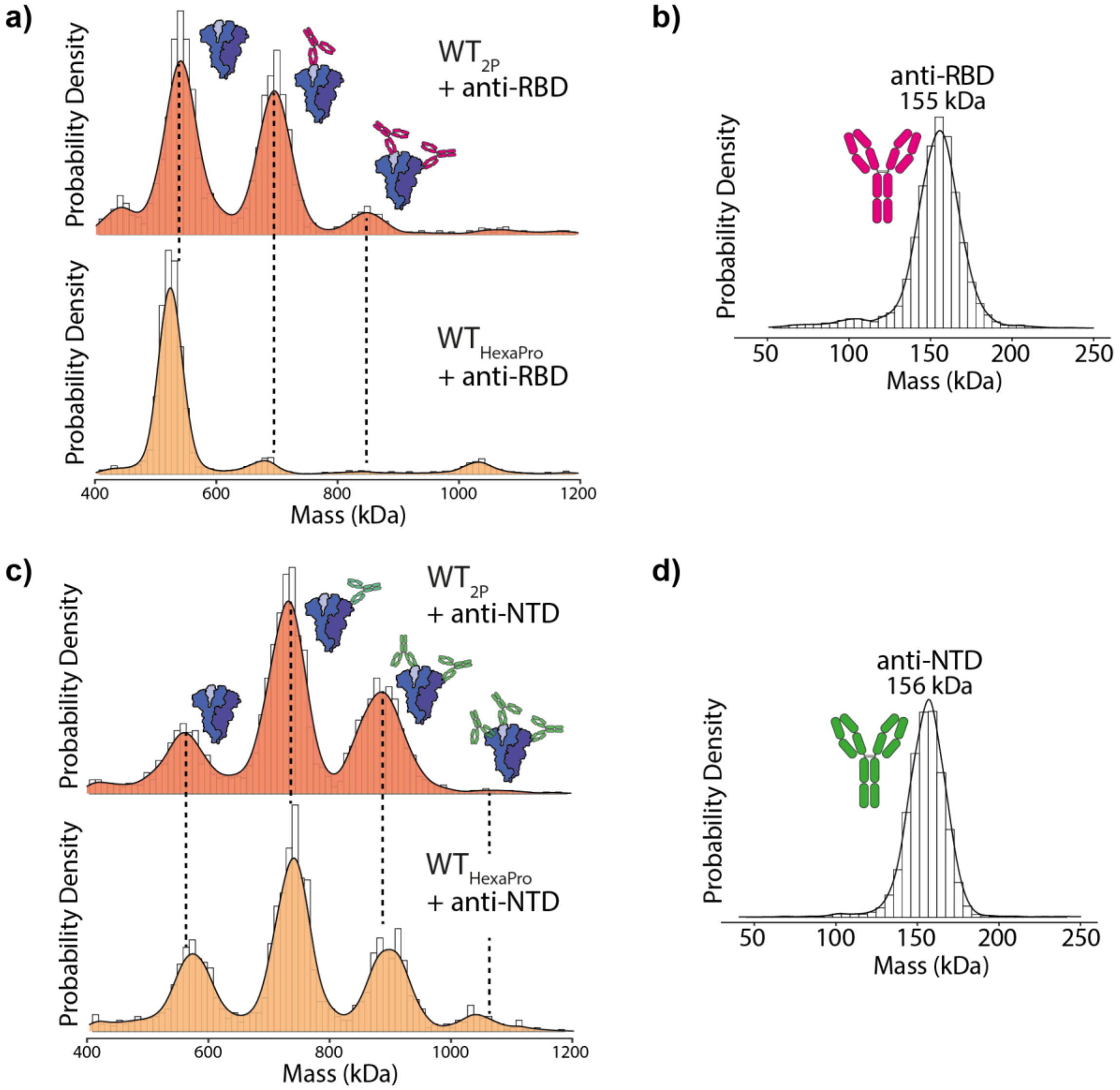

Figure S5. MP of WT 2P and HexaPro with NTD and RDB targeting antibody. **(a)** Binding between WT 2P and HexaPro (25 nM) spike with an N-terminal domain (NTD) targeting antibody (100 nM). Representative histograms with overlaid kernel density estimates are shown. **(b)** Mass photometry of NTD antibody alone. **(c)** Binding between WT 2P and HexaPro (25 nM) spike with a receptor-binding domain (RBD) targeting antibody (50 nM). Representative histograms with overlaid kernel density estimates are shown. **(d)** Mass photometry of RBD antibody alone.

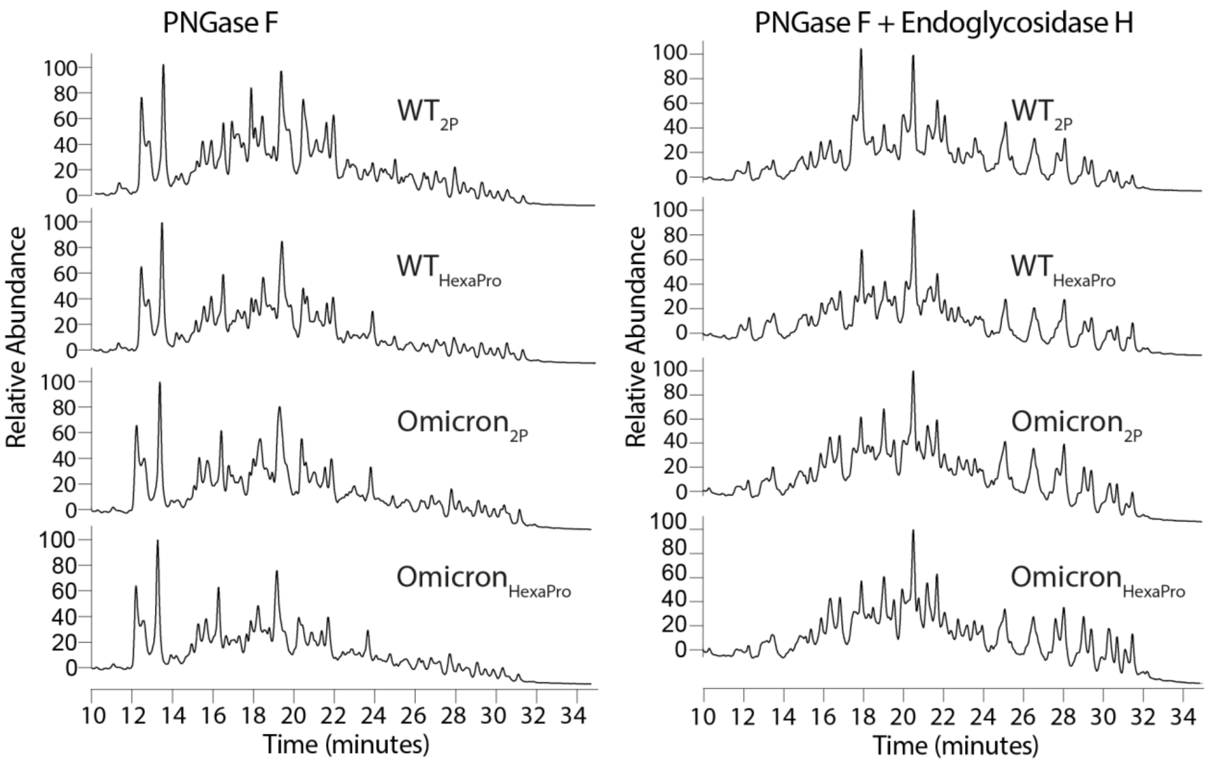

Figure S6. N-glycan UHPLC chromatograms. HILIC-UHPLC of N-glycans labelled with 2-aminobenzoic acid (2-AA). Left column are intact glycans (PNGase F treated). PNGase F and Endoglycosidase H treated N-glycans are shown on the right and show the extent of oligomannose and hybrid structures.

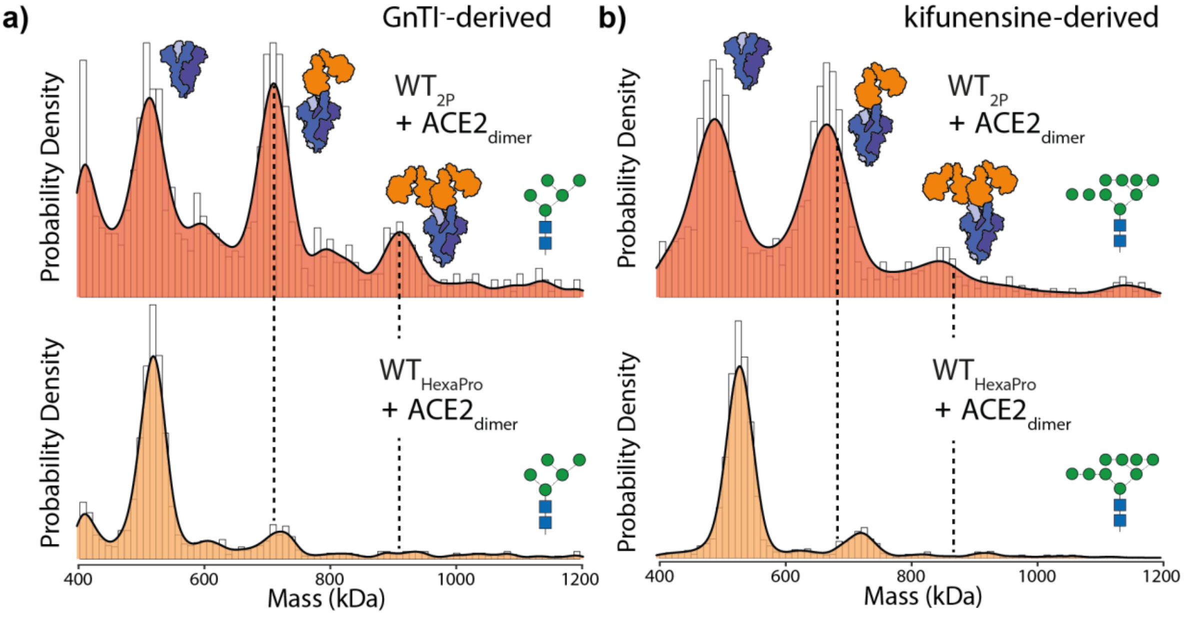

Figure S7. MP of glycoengineered spikes with dimeric ACE2. ACE2 dimers plus WT 2P and HexaPro spikes from (**a**) N-acetylglucosaminyltransferase I ($GnTI^{-}$) deficient cells (HEK293S) or spikes expressed in the presence of (**b**) kifunensine (kif). GnTI- spikes result in $Man_5GlcNAc_2$ and kif spikes result in $Man_9GlcNAc_2$ N-glycans at all N-glycosylation sites (cartoon glycan structures are shown). MP measurements are with 25 nM spike and 100 nM dimeric ACE2.

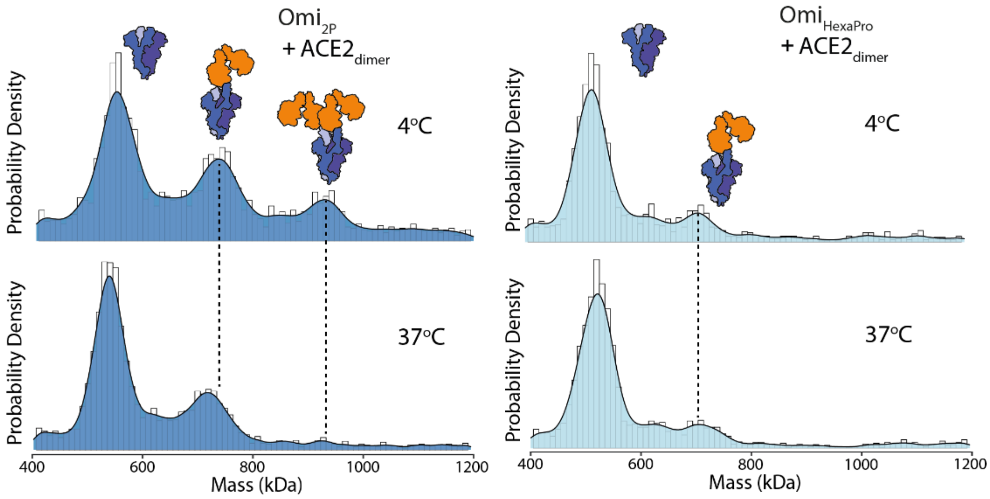

Figure S8. Dimeric ACE2 binding to temperature treated spikes. 2P and HexaPro Omicron spikes were incubated on ice or at 37°C prior to the addition of dimeric ACE2 (25 nM spike and 100 nM ACE2). Representative histograms with overlaid kernel density estimates are shown.

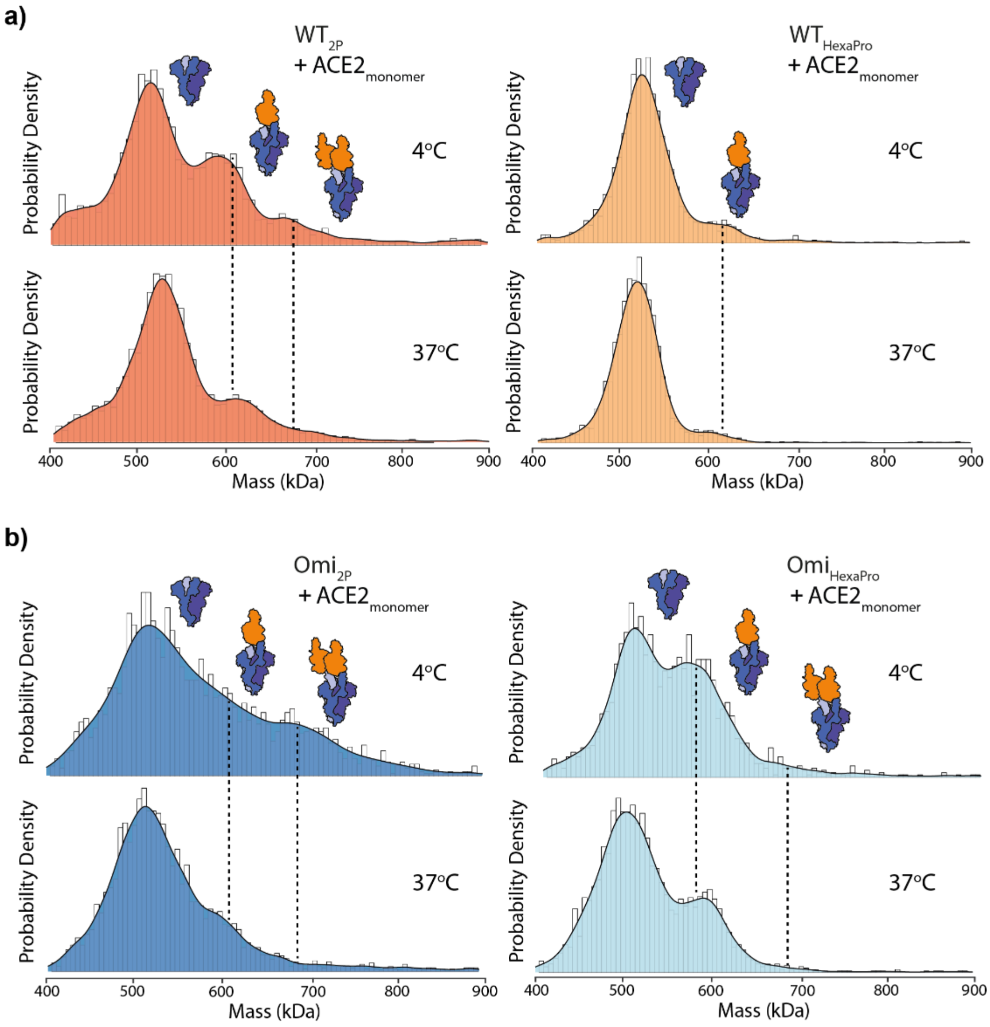

Figure S9. Monomeric ACE2 binding to temperature treated spikes. MP of (**a**) WT and (**b**) Omicron spike (25 nM) plus 100 nM ACE2 monomer. Spikes were preincubated on ice or at 37°C prior to the addition of monomeric ACE2. Representative histograms with overlaid kernel density estimates are shown.

4. Supplementary Tables

Table S1. MP of non-stabilised, 2P and HexaPro Wuhan spike. Trimeric distribution of WT non-stabilised, 2P and HexaPro spikes measured by MP. Monomer, dimer and trimer counts are calculated in relation to total spike counts.

	Monomer	Dimer	Trimer
$WT_{non\text{-}stablised}$	43.7%	12.2%	44.1%
WT_{2P}	18.6%	11.1%	70.3%
$WTH_{HexaPro}$	7.2%	2.3%	90.5%

.

Table S2. ACE2 dimer titration MP of 2P and HexaPro spike. Interaction between spikes and dimeric ACE2 measured by MP. The interaction is represented as % total occupancy, which was calculated using the sum of all spike counts including species with 0, 1, 2 and 3 ACE2 molecules bound and expressed as a percentage of 1, 2 and 3 ACE2 bound counts compared to total spike counts.

	100nM	80nM	60nM	40nM	20nM
WT_{2P}	53.2%	43.4%	35.1%	21.1%	11.9%
$WT_{HexaPro}$	25.7%	20.9%	17.1%	10.6%	5.0%
Omi_{2P}	46.3%	32.8%	22.3%	21.8%	13.3%
$Omi_{HexaPro}$	32.6%	19.8%	12.8%	12.2%	5.5%

Table S3. ACE2 monomer titration MP of 2P and HexaPro spike. Interaction between spikes and monomeric ACE2 measured by MP. The interaction is represented as % total occupancy, which was calculated using the sum of all spike counts including species with 0, 1, 2 and 3 ACE2 molecules bound and expressed as a percentage of 1, 2 and 3 ACE2 bound counts compared to total spike counts.

	100nM	80nM	60nM	40nM	20nM
WT_{2P}	43.1%	36.2%	34.4%	21.2%	14.3%
$WT_{HexaPro}$	29.0%	23.0%	15.2%	10.4%	4.6%
Omi_{2P}	58.3%	54.4%	50.7%	35.0%	21.6%
$Omi_{HexaPro}$	52.1%	39.1%	39.1%	37.1%	21.0%

Table S4. WT N-glycan UHPLC quantitation. Integrated glycan peak areas were normalised to peak labelled 18 across each spike preparation and plotted as relative percentages. Fold changes were calculated between WT 2P and HexaPro spikes.

	Peak Area		Normalised Peak Area		
Peak ID	WT_{2P}	$WT_{HexaPro}$	WT_{2P}	$WT_{HexaPro}$	Fold change
1	456073	393427	6.22	6.34	0.02
2	2273065	2507795	31.02	40.43	0.30
3	4801676	5248449	65.53	84.61	0.29
4	1000606	1427623	13.66	23.01	0.69
5	1192211	982965	16.27	15.85	-0.03
6	5702897	6565682	77.83	105.85	0.36
7	1980904	2101739	27.03	33.88	0.25
8	3362344	1950278	45.89	31.44	-0.31
9	9700540	7802178	132.39	125.78	-0.05
10	1760832	1398574	24.03	22.55	-0.06
11	2872356	3309902	39.20	53.36	0.36
12	2555322	3306734	34.87	53.31	0.53
13	2061066	1707913	28.13	27.53	-0.02
14	3266730	2193576	44.58	35.36	-0.21
15	853490	580706	11.65	9.36	-0.20
16	428484	1548837	5.85	24.97	3.27
17	388235	249890	5.30	4.03	-0.24
18	7327324	6203025	100.00	100.00	0.00

Table S5. Omicron N-glycan UHPLC quantitation. Integrated glycan peak areas were normalised to peak labelled 18 across each spike preparation and plotted as relative percentages. Fold changes were calculated between Omicron (Omi) 2P and HexaPro spikes.

	Peak Area		Normalised Peak Area		
Peak ID	Omi_{2P}	$Omi_{HexaPro}$	Omi_{2P}	$Omi_{HexaPro}$	Fold change
1	231983	180446	5.30	3.27	-0.38
2	1944204	1700419	44.44	30.84	-0.31
3	3757325	3517724	85.88	63.80	-0.26
4	918776	849433	21.00	15.40	-0.27
5	522639	583395	11.95	10.58	-0.11
6	3827955	3897623	87.50	70.69	-0.19
7	1635775	1759879	37.39	31.92	-0.15
8	1191259	1094001	27.23	19.84	-0.27
9	4540668	3998826	103.79	72.52	-0.30
10	567250	924664	12.97	16.77	0.29
11	2067755	2095143	47.26	38.00	-0.20
12	2348178	2115610	53.67	38.37	-0.29
13	1355913	1371882	30.99	24.88	-0.20
14	1777431	1769350	40.63	32.09	-0.21
15	873727	595191	19.97	10.79	-0.46
16	1384775	774381	31.65	14.04	-0.56
17	290116	234601	6.63	4.25	-0.36
18	4375028	5514049	100.00	100.00	0.00

Table S6. WT 2P site-specific glycan quantification. Extracted ion chromatogram (XIC) intensities for each glycopeptide were summed for each glycan class, calculated relative to total summed ion intensities of all glycopeptide ions and expressed as a percentage.

	N165		N234		N343	
	XIC	%	XIC	%	XIC	%
Unoccupied						
Man9	7.24E+07	0.16%	4.02E+09	9.55%		
Man8			1.66E+10	39.32%		
Man7			8.43E+09	20.02%		
Man6	1.58E+08	0.35%	2.8E+09	6.66%		
Man5	7.65E+09	16.78%	4.18E+09	9.93%	9.93E+08	2.29%
hybrid	8.02E+08	1.76%	9.26E+08	2.20%	2.23E+09	5.14%
Mono	3.28E+08	0.72%	1.96E+08	0.46%	1.51E+09	3.48%
Bi	1.45E+10	31.84%	1.33E+09	3.15%	1.96E+10	45.27%
Tri	2.02E+10	44.31%			1.57E+10	36.23%
Tetra	1.04E+09	2.29%	3.67E+09	8.71%	32700000	0.08%

Table S7. WT HexaPro site-specific glycan quantification. Extracted ion chromatogram (XIC) intensities for each glycopeptide were summed for each glycan class, calculated relative to total summed ion intensities of all glycopeptide ions and expressed as a percentage.

	N165		N234		N343	
	XIC	%	XIC	%	XIC	%
Unoccupied						
Man9			3.5E+09	73.30%		
Man8			9.87E+08	20.70%		
Man7			2.57E+08	5.38%		
Man6	1.25E+07	0.56%	2.94E+07	0.62%		
Man5	1.37E+09	61.42%				
hybrid	5.90E+07	2.65%			7.88E+07	3.52%
Mono					3.85E+07	1.72%
Bi	2.97E+08	13.35%			1.56E+09	69.85%
Tri	4.54E+08	20.40%			5.57E+08	24.90%
Tetra	1.09E+07	0.49%				

Table S8. Omicron 2P site-specific glycan quantification. Extracted ion chromatogram (XIC) intensities for each glycopeptide were summed for each glycan class, calculated relative to total summed ion intensities of all glycopeptide ions and expressed as a percentage.

	N165		N234		N343	
	XIC	%	XIC	%	XIC	%
Unoccupied						
Man9			2.68E+09	58.58%		
Man8			5.87E+07	1.28%		
Man7			1.30E+08	2.84%		
Man6	1.27E+09	13.66%	7.75E+07	1.69%		
Man5	6.48E+09	69.65%	1.52E+07	0.33%		
hybrid	3.03E+08	3.25%	1.49E+07	0.33%		
Mono	1.39E+08	1.49%				
Bi	5.65E+08	6.08%	4.41E+08	9.62%	3.01E+08	8.16%
Tri	3.26E+08	3.50%			3.09E+09	83.96%
Tetra	2.2E+08	2.36%	5.01E+08	10.94%	2.9E+08	7.88%

Table S9. Omicron HexaPro site-specific glycan quantification. Extracted ion chromatogram (XIC) intensities for each glycopeptide were summed for each glycan class, calculated relative to total summed ion intensities of all glycopeptide ions and expressed as a percentage.

	N165		N234		N343	
	XIC	%	XIC	%	XIC	%
Unoccupied						
Man9			1.15E+10	75.44%		
Man8			2.90E+09	19.03%		
Man7			4.51E+08	2.96%		
Man6	5.96E+08	12.14%	2.35E+08	1.54%		
Man5	3.64E+09	74.20%	7.79E+07	0.51%		
hybrid	1.41E+08	2.87%				
Mono						
Bi	4.38E+08	8.92%			2.58E+08	20.60%
Tri	2.96E+07	0.60%			9.94E+08	79.40%
Tetra	6.17E+07	1.26%	8.09E+07	0.53%		

Table S10. The effect of temperature upon spike ACE2 binding. Interaction between spikes and ACE2 is represented as % total occupancy, which was calculated using the sum of all spike counts including species with 0, 1, 2 and 3 ACE2 molecules bound and expressed as a percentage of 1, 2 and 3 ACE2 bound counts compared to total spike counts. Fold changes were calculated between 4°C and 37°C for HexaPro and 2P inclusively.

	$ACE2_{monomer}$			$ACE2_{dimer}$		
	4°C	37°C	Fold change	4°C	37°C	Fold change
WT_{2P}	47.0%	18.1%	1.6	41.2%	23.0%	0.8
$WT_{hexaPro}$	13.0%	6.2%	1.1	13.6%	7.4%	0.8
Omi_{2P}	58.3%	27.5%	1.1	50.7%	33.4%	0.5
$Omi_{hexaPro}$	58.9%	32.4%	0.8	16.3%	13.6%	0.2

Mean fold change HexaPro = 0.7 ((1.1+0.8+0.8+0.2)/4)
Mean fold change 2P = 1.0 ((1.6+0.8+1.1+0.5)/4)
Mean occupancy (37°C) HexaPro = 15% ((6.2+32.4+7.4+13.6)/4)
Mean occupancy (37°C) 2P = 26% ((18.1+27.5+23+33.4)/4)

5. References

1. S. M. Gobeil, K. Janowska, S. McDowell, K. Mansouri, R. Parks, K. Manne, V. Stalls, M. F. Kopp, R. Henderson, R. J. Edwards, B. F. Haynes and P. Acharya, *Cell Rep*, 2021, **34**, 108630.
2. J. Brun, S. Vasiljevic, B. Gangadharan, M. Hensen, V. C. A, M. L. Hill, J. L. Kiappes, R. A. Dwek, D. S. Alonzi, W. B. Struwe and N. Zitzmann, *ACS Cent Sci*, 2021, **7**, 586-593.
3. C. L. Hsieh, J. A. Goldsmith, J. M. Schaub, A. M. DiVenere, H. C. Kuo, K. Javanmardi, K. C. Le, D. Wrapp, A. G. Lee, Y. Liu, C. W. Chou, P. O. Byrne, C. K. Hjorth, N. V. Johnson, J. Ludes-Meyers, A. W. Nguyen, J. Park, N. Wang, D. Amengor, J. J. Lavinder, G. C. Ippolito, J. A. Maynard, I. J. Finkelstein and J. S. McLellan, *Science*, 2020, **369**, 1501-1505.
4. J. M. Schaub, C. W. Chou, H. C. Kuo, K. Javanmardi, C. L. Hsieh, J. Goldsmith, A. M. DiVenere, K. C. Le, D. Wrapp, P. O. Byrne, C. K. Hjorth, N. V. Johnson, J. Ludes-Meyers, A. W. Nguyen, N. Wang, J. J. Lavinder, G. C. Ippolito, J. A. Maynard, J. S. McLellan and I. J. Finkelstein, *Nat Protoc*, 2021, **16**, 5339-5356.
5. F. Soltermann, E. D. B. Foley, V. Pagnoni, M. Galpin, J. L. P. Benesch, P. Kukura and W. B. Struwe, *Angew Chem Int Ed Engl*, 2020, **59**, 10774-10779.
6. D. S. Alonzi, D. C. Neville, R. H. Lachmann, R. A. Dwek and T. D. Butters, *Biochem J*, 2008, **409**, 571-580.
7. J. A. Vizcaíno, E. W. Deutsch, R. Wang, A. Csordas, F. Reisinger, D. Ríos, J. A. Dianes, Z. Sun, T. Farrah, N. Bandeira, P. A. Binz, I. Xenarios, M. Eisenacher, G. Mayer, L. Gatto, A. Campos, R. J. Chalkley, H. J. Kraus, J. P. Albar, S. Martinez-Bartolomé, R. Apweiler, G. S. Omenn, L. Martens, A. R. Jones and H. Hermjakob, *Nat Biotechnol*, 2014, **32**, 223-226.
